# Feasibility of a streamlined tuberculosis diagnosis and treatment initiation strategy

**DOI:** 10.5588/ijtld.16.0699

**Published:** 2017-07-01

**Authors:** P. B. Shete, T. Nalugwa, K. Farr, C. Ojok, M. Nantale, P. Howlett, P. Haguma, E. Ochom, F. Mugabe, M. Joloba, L. H. Chaisson, D. W. Dowdy, D. Moore, J. L. Davis, A. Katamba, A. Cattamanchi

**Affiliations:** * Division of Pulmonary and Critical Care Medicine, University of California San Francisco and Zuckerberg San Francisco General Hospital, San Francisco; † Curry International Tuberculosis Center, University of California San Francisco, San Francisco, California, USA; ‡ School of Medicine, Makerere University College of Health Sciences, Kampala, Uganda; § Faculty of Infectious and Tropical Diseases and TB Centre, London School of Hygiene and Tropical Medicine, London, UK; ¶ Uganda National Tuberculosis and Leprosy Control Programme, Kampala; # School of Biomedical Sciences, Makerere University College of Health Sciences, Kampala, Uganda; ** Department of Epidemiology, Johns Hopkins Bloomberg School of Public Health, Baltimore, Maryland; †† Department of Epidemiology of Microbial Diseases, Yale School of Public Health, New Haven, Connecticut; ‡‡ Section of Pulmonary, Critical Care, and Sleep Medicine, Yale School of Medicine, New Haven, Connecticut, USA

**Keywords:** tuberculosis, diagnosis, implementation research, Xpert

## Abstract

**OBJECTIVE:**

To assess the feasibility of a streamlined strategy for improving tuberculosis (TB) diagnostic evaluation and treatment initiation among patients with presumed TB.

**DESIGN:**

Single-arm interventional pilot study at five primary care health centers of a streamlined, SIngle-saMPLE (SIMPLE) TB diagnostic evaluation strategy: 1) examination of two smear results from a single spot sputum specimen using light-emitting diode fluorescence microscopy, and 2) daily transportation of smear-negative sputum samples to Xpert® MTB/RIF testing sites.

**RESULTS:**

Of 1212 adults who underwent sputum testing for TB, 99.6% had two smears examined from the spot sputum specimen. Sputum was transported for Xpert testing within 1 clinic day for 83% (907/1091) of the smear-negative patients. Of 157 (13%) patients with bacteriologically positive TB, 116 (74%) were identified using sputum smear microscopy and 41 (26%) using Xpert testing of smear-negative samples. Anti-tuberculosis treatment was initiated in 142 (90%) patients with bacteriologically positive TB, with a median time to treatment of 1 day for smear-positive patients and 6 days for smear-negative, Xpert-positive patients.

**CONCLUSION:**

The SIMPLE TB strategy led to successful incorporation of Xpert testing and rapid treatment initiation in the majority of patients with bacteriologically confirmed TB in a resource-limited setting.

## RESUME

**OBJECTIF:**

Evaluer la faisabilité d'une stratégie simplifiée pour améliorer l'évaluation du diagnostic et la mise en route du traitement de la tuberculose (TB) parmi les patients avec une présomption de TB.

**SCHÉMA:**

Étude pilote d'intervention à un seul bras dans cinq centres de soins de santé primaires d'une stratégie simplifiée, l'évaluation du diagnostic de TB sur un seul échantillon, SIngle-saMPLE (SIMPLE) : 1) examen de deux résultats de frottis d'un seul échantillon de crachats grâce à un microscope en fluorescence à LED, et 2) transport quotidien d'échantillons de crachats à frottis négatif vers les sites de test Xpert® MTB/RIF.

**RÉSULTATS:**

Sur 1212 adultes qui ont eu un recueil de crachats pour un test de TB, 99,6% ont bénéficié de deux frottis de l'échantillon de crachats examiné. Les crachats ont été transportés pour le test Xpert en moins d'un jour pour 83% (907/1091) des patients à frottis négatif. Sur 157 (13%) patients avec une TB à bactériologie positive, 116 (74%) ont été identifiés par microscopie de frottis de crachats et 41 (26%) par un test Xpert sur des échantillons à frottis négatif. Le traitement de TB a été initié chez 142 (90%) patients atteints de TB à bactériologie positive, avec un délai médian de traitement d'un jour pour les patients à frottis positif et de 6 jours pour les patients à frottis négatif, Xpert positif.

**CONCLUSION:**

La stratégie TB SIMPLE a abouti à une incorporation réussie du test Xpert et à une mise en route rapide du traitement chez la majorité des patients atteints de TB confirmée par bactériologie dans un contexte aux ressources limitées.

## RESUMEN

**OBJETIVO:**

Evaluar la factibilidad de dinamizar la estrategia de diagnóstico, evaluación e iniciación del tratamiento de los pacientes con presunción clínica de la tuberculosis (TB).

**MÉTODO:**

Se llevó a cabo un estudio preliminar de intervención con un solo grupo experimental en cinco centros de atención primaria, sobre la dinamización de la estrategia SIngle-saMPLE (SIMPLE) de evaluación y diagnóstico de la TB, que comportaba: 1) el examen con microscopio LED de fluorescencia de los resultados de dos baciloscopias a partir de una muestra inmediata única; y 2) el transporte diario de las muestras con baciloscopia negativa a otro centro con el fin de practicar la prueba Xpert® MTB/RIF.

**RESULTADOS:**

De los 1212 adultos en quienes se recogieron muestras de esputo para la investigación de la TB, en el 99,6% se examinaron dos frotis de una muestra inmediata. Las muestras se transportaron en el día de la consulta con el fin de practicar la prueba Xpert en el 83% de los pacientes con baciloscopia negativa (907/1091). De los 157 pacientes con TB confirmada bacteriológicamente (13%), 116 se detectaron mediante el examen microscópico del frotis (74%) y 41 mediante la prueba Xpert en las muestras con baciloscopia negativa (26%). El tratamiento de la TB en 142 pacientes con confirmación bacteriológica (90%) se inició en un lapso mediano de un día en los pacientes con baciloscopia positiva y 6 días en los pacientes con baciloscopia negativa y prueba Xpert positiva.

**CONCLUSIÓN:**

La estrategia SIMPLE de detección de la TB facilitó la incorporación eficaz de la prueba Xpert y la rápida iniciación del tratamiento en la mayoría de los pacientes con TB confirmada bacteriológicamente, en un entorno de escasos recursos.

PROMPT DIAGNOSIS AND TREATMENT of tuberculosis (TB) patients is essential for making progress towards TB elimination. However, 4.3 million of the estimated 10.4 million new cases in 2016 were not detected and reported to the World Health Organization (WHO).^1^ There are three overarching explanations for this large gap: TB patients are not being notified to public health authorities, they are not seeking care, or they are not being diagnosed and treated even after seeking care. The last explanation represents a clear health system failure that is common in high-burden countries: a recent systematic review found that up to 38% of sputum smear-positive patients in Africa are lost to follow-up before treatment initiation.^2^ Patients with smear-negative TB are even less likely to complete a full diagnostic evaluation and be linked to treatment.

A principal reason for these failures with regard to care is the inadequacy of the current approach of using smear microscopy for TB diagnosis at primary health centers. First, smear microcopy has suboptimal sensitivity, identifying only about 50% of patients who actually have TB.^3^ Second, the typical process of sputum collection and smear examination is burdensome for patients. It requires multiple visits over multiple days for testing, the receipt of results, starting treatment or undergoing further work-up, and consumption of up to 3 months of household income.^4^–^8^ It is not surprising that a substantial proportion of patients do not complete this drawn-out diagnostic process.^9^–^12^

To address these limitations, there has been substantial donor investment in the scale-up of Xpert^®^ MTB/RIF (Cepheid, Sunnyvale, CA, USA), a novel semi-automated molecular assay endorsed by the WHO in 2010.^13^ Xpert identifies 90% of TB cases within 2 h, and can be performed with minimal human resource requirements.^14^ High cost and infrastructure requirements, however, mean that the majority of Xpert devices are being placed at district or higher-level facilities^13^ accessed by <15% of the population.^15^ A cluster-randomized trial found that current Xpert implementation did not impact mortality; the main reason was the failure to link patients with confirmed TB to treatment.^16^ Strategies for improving referral to Xpert testing from peripheral microscopy centers are essential to achieve access to, utilization of and impact of Xpert on patient and public health outcomes.

There is increasing evidence that behavioral theory and implementation frameworks are useful for identifying and targeting barriers to uptake of guidelines.^17^ We previously conducted theory-informed, mixed-methods studies to identify key patient- and provider-level barriers to the uptake of TB evaluation guidelines at health centers in Uganda.^18^–^20^ Based on this formative assessment, we identified two intervention components with strong potential to improve guideline-based TB diagnosis: 1) restructuring of clinic-level procedures via single-sample (one specimen, two smears) light-emitting diode (LED) fluorescence microscopy (FM),^21^ and 2) daily sputum transport of smear-negative samples and all human immunodeficiency virus (HIV) positive samples to Xpert testing sites. Together these interventions, known as the SIngle saMPLE (SIMPLE) TB evaluation strategy, are intended to reduce the burden of sputum smear evaluation and Xpert testing on patients and providers through a combination of behavior change and service delivery interventions that are feasible within the local context.

In this pilot study, we introduced the SIMPLE TB strategy at five health centers in Uganda. Our objective was to evaluate the feasibility and implementation of each component.

## METHODS

### Study setting and population

This single-arm interventional pilot study was conducted at five peripheral health centers (level IV) government health centers in rural Uganda. Level IV health centers are the lowest level of the health system where TB diagnosis (microscopy) and treatment are available, covering a population of approximately 100 000. These services are provided free-of-charge through the National Tuberculosis and Leprosy Programme (NTLP). Health centers were included in the pilot if they were 1) using standard (multi-day) sputum smear microscopy as the primary method of TB diagnosis; 2) participating in NTLP-sponsored external quality assurance (EQA) for sputum smear microscopy; and 3) affiliated with a district or regional hospital offering Xpert testing. We collected data on consecutive adults who were screened for chronic cough (>2 weeks) by the health center and who had sputum collected for TB diagnosis after the introduction of the two SIMPLE TB strategy components at each health center. Mycobacterial culture and speciation were not routinely performed, as they are not standard of care for TB diagnosis at the peripheral health centers.

The study protocol was approved by the institutional review boards of the University of California San Francisco, San Francisco, CA, USA, and Makerere University, Kampala, and by the Uganda National Council for Science and Technology, Kampala, Uganda.

### Study procedures

#### Introduction of interventions

**Figure 1.**
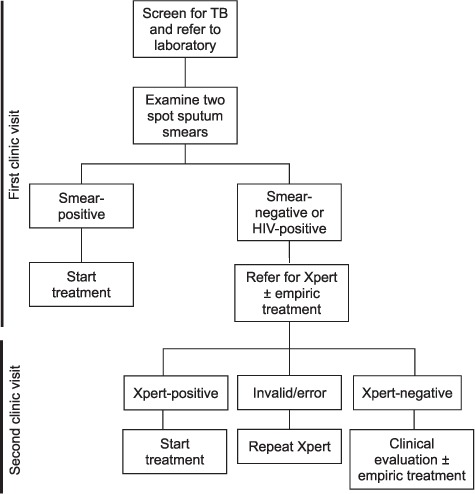
SIMPLE TB diagnostic algorithm. The SIMPLE TB algorithm calls for two smears from the spot sputum sample to be prepared and examined. Patients with a positive sputum smear should be initiated on treatment during their initial visit to the health center. Patients with two negative smears or with HIV infection should have the remainder of the spot sputum sample sent for Xpert testing. Patients with positive Xpert results should be started on treatment at their next visit to the health center. TB = tuberculosis; HIV = human immunodeficiency virus; SIMPLE = SIngle-saMPLE.

SIMPLE TB strategy components were introduced at each health center during a 2-day site visit conducted by research staff and NTLP staff. The health center laboratory was provided with a Primo Star iLED microscope (Zeiss Microscopy, Jena, Germany), and laboratory officers were trained to perform LED FM using the Uganda National Tuberculosis Reference Laboratory (NTRL) training manual. Proficiency in LED FM was checked by completion of a post-training evaluation form and by practical examination by senior laboratory technologists who are members of the research team. During the study period, EQA for LED FM was performed on a monthly basis by study staff and on a quarterly basis by Uganda NTRL staff. A meeting was held with clinical, laboratory and pharmacy staff to discuss redesigning the TB microscopy process to facilitate same-day diagnosis and treatment of smear-positive TB. Key process changes included screening for cough at the time of patient registration, immediate referral of patients with chronic cough to the laboratory for sputum collection before the clinician visit, on-demand processing of sputum to prepare and analyze two smears rather than batch processing, and training additional staff to initiate TB treatment rather than relying on a single TB pharmacist (Figure 1). Transportation of residual sputum from smear-negative samples and HIV-positive patients from study health centers to Xpert referral hubs was arranged by motorcycle (*boda boda*) on a daily, rather than weekly, basis. Patients were instructed to return the following day to collect Xpert results. After the initial 2-day visit, study staff visited the health centers every month to reinforce training messages and adherence to the intervention strategy.

#### Data collection

We extracted patient demographic and clinical data monthly from routine health center records, including laboratory and treatment registers, microscopy and Xpert requisition forms, and GeneXpert software. Individual patient records were matched across data sources manually. Discrepancies were resolved through discussion with health center staff. De-identified patient data were entered into a secure REDCap (Research Electronic Data Capture; Vanderbilt University, Nashville, TN, USA) database.^22^ Data entered included HIV status, dates and results of sputum smear examination, dates of sputum transport to an Xpert testing facility, dates and results of Xpert testing, and date of TB treatment initiation. Smear- or Xpert-positive patients not recorded as having started treatment >6 months after initial sputum collection were contacted by telephone to determine whether they had started treatment elsewhere; for those who had, the information was verified with the health center where the patient was registered for treatment.

### Outcomes

Outcomes for the present study were based on process metrics that reflect implementation of the SIMPLE TB algorithm components: numbers and proportions of patients referred for TB testing who had two smears examined from the spot specimen, smear-positive patients who initiated anti-tuberculosis treatment, smear-negative patients who had their sputum sample referred for Xpert testing, and Xpert-positive patients who initiated anti-tuberculosis treatment. Additional outcomes included the time-to-diagnosis and time-to-treatment of smear-positive and Xpert-positive patients.

### Data analysis

We described patient characteristics and the fidelity of process implementation using proportions with 95% confidence intervals (CIs) for dichotomous outcomes and either medians with interquartile ranges (IQRs) or means with standard deviations (SDs) for continuous outcomes. We compared differences in proportions across sites using the χ^2^ test of proportions. We performed all analyses using Stata, version 14 (Stata Corporation, College Station, TX, USA).

## RESULTS

### Demographic and clinical characteristics

Of 1212 adults (Table 1) who underwent sputum testing for TB between February 2015 and April 2016, 684 (56%) were female; the median age was 40 years (IQR 30–50). HIV infection was documented in 647 (53%) patients. Patient demographics and clinical characteristics were similar across study sites.

**Table 1:**
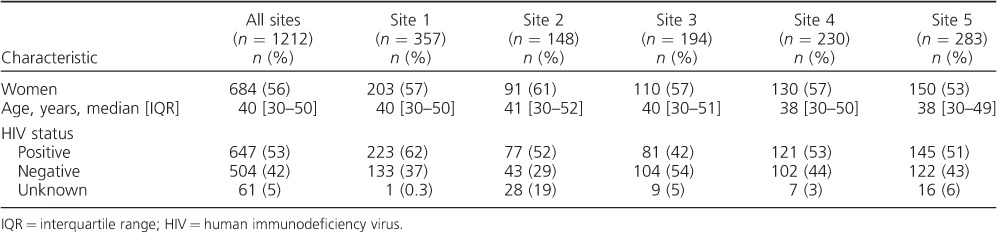
Demographic and clinical characteristics of the study group

### Testing for tuberculosis

**Figure 2.**
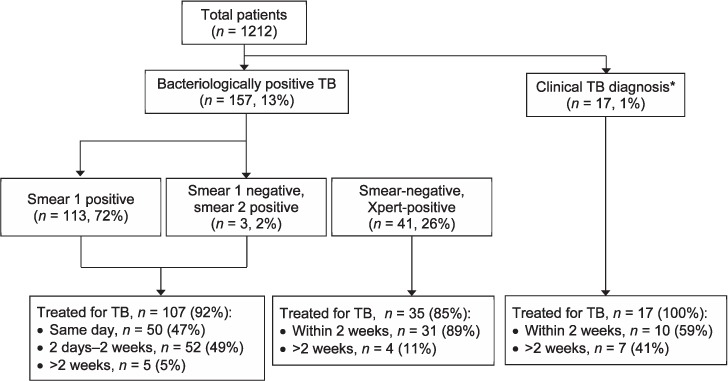
Overall tuberculosis diagnosis and treatment. The diagram describes the study flow and outcomes for patients using the SIMPLE strategy. Of 1212 patients evaluated for TB using the SIMPLE approach, 157 (13%) were microbiologically diagnosed. The majority of patients with bacteriological diagnosis were started on treatment (90%); however, same-day treatment was initiated in less than half of patients. *Treated for TB in the absence of a positive smear or Xpert result. TB = tuberculosis; SIMPLE = SIngle-saMPLE.

Overall, 157 of 1212 (13%) patients had microbiologically confirmed (i.e., smear- and/or Xpert-positive) TB (Figure 2). Site-specific prevalence of bacteriologically positive TB varied from 7% to 17%. The first smear result from the spot specimen was positive in 113 (9.3%) patients, and the second smear of the spot specimen identified three additional smear-positive patients (116 positive smears) (incremental yield 2.6%, 95%CI 0.5–7.4). Xpert testing was performed for 995/1091 (91%) smear-negative patients (median time to testing 1 day, IQR 0–2), and 41 additional patients with TB were identified (incremental yield 26.1%, 95%CI 19.4–33.7). Rifampin (RMP) resistance was not detected in any of these smear-negative patients. Of all smear-negative patients tested using Xpert, 61 (6.1%) had indeterminate/invalid results. Xpert testing was also performed for 26/54 (48.1%) smear-positive patients with documented HIV infection (median time to testing 1 day, IQR 0–2): one was identified with RMP resistance (one patient had an invalid/indeterminate result).

### Anti-tuberculosis treatment

Of 157 patients with bacteriologically positive TB, 142 (90.4%) were initiated on anti-tuberculosis treatment (Figure 2): 107/116 (92.2%) patients diagnosed using sputum smear microscopy and 35/41 (85.4%) patients diagnosed using Xpert. Among those treated, the median time to treatment was 1 day (IQR 0–1) for smear-positive patients and 6 days (IQR 2–11) for smear-negative, Xpert-positive patients. In addition, 17 patients were initiated on treatment in the absence of a positive microbiological test, with a median time to treatment of 4 days (IQR 0–249).

### Adherence to the SIMPLE TB algorithm

**Figure 3.**
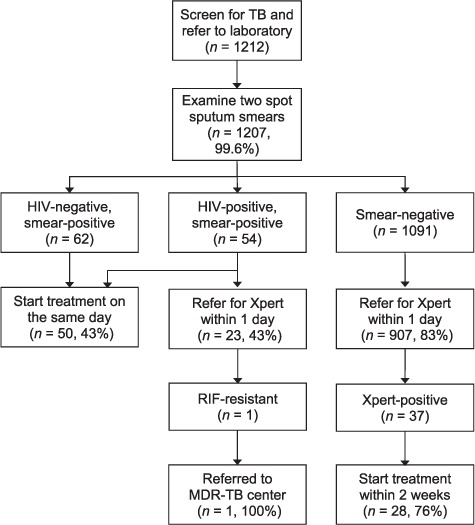
Adherence to the SIMPLE TB diagnostic algorithm. The diagram describes the flow of patients through the SIMPLE algorithm. Almost all patients referred for testing had two spot sputum smears examined, with results. Same-day treatment initiation for smear-positive patients and Xpert referral for HIV-positive patients had moderate fidelity of implementation (43%). The majority of smear-negative patients (83%) were referred for Xpert testing in 1 day. These results suggest that a facilitated TB diagnostic approach is feasible, particularly for smear-negative patients. TB = tuberculosis; HIV = human immunodeficiency virus; RIF = rifampin; MDR-TB = multidrug-resistant TB; SIMPLE = SIngle-saMPLE.

Two smears were prepared and examined from the spot sputum specimens of 1207 (99.6%) patients (Figure 3). Of the 116 smear-positive patients, 50 (43.1%) started treatment at the initial health center visit. The remaining sputum was referred for Xpert testing within 1 clinic day for 907/1091 (83.1%) sputum smear-negative patients. Of the 37 patients who had a positive Xpert result, 28 (75.7%) started anti-tuberculosis treatment within 2 weeks of their initial health center visit. In addition, 23/54 (42.6%) smear-positive patients with documented HIV infection had sputum referred for Xpert testing within 1 clinic day, in accordance with Xpert implementation guidelines. One of these patients had RMP resistance and was referred successfully to a treatment center for multidrug-resistant TB.

There was some variability in adherence to the SIMPLE TB algorithm across sites (Table 2). Comparison between sites showed that two smears were examined in 98.6–100% of patients (*P* = 0.24). For smear-negative patients, sputum was transported to the Xpert testing site within 1 clinic day for 77–88% of smear-negative patients (*P* = 0.002) and 17–67% of smear-positive patients with documented HIV infection (*P* = 0.28). Anti-tuberculosis treatment was initiated at the initial health center visit for 16–73% of smear-positive patients (*P* = 0.01) and initiated within 2 weeks for 60–83% of smear-negative, Xpert-positive patients (*P* = 0.56).

**Table 2:**
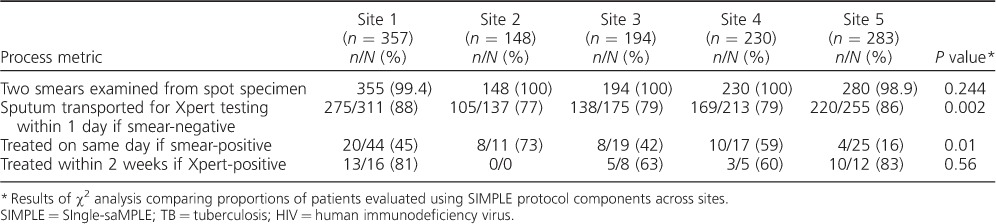
Site-level variation in implementation of the SIMPLE TB algorithm

## DISCUSSION

To reduce the burden of TB, strategies are needed to facilitate the uptake of new diagnostics and ensure that patients diagnosed with TB are linked to treatment. In this pilot study, we evaluated a streamlined TB diagnostic strategy that sought to reduce the burden of diagnostic evaluations for TB on patients and providers. We found that single-sample LED FM and daily transport of smear-negative samples to Xpert testing facilities are feasible in a resource-limited setting with high TB prevalence. Nearly all patients had two smears examined, and 91% of smear-negative patients underwent sputum testing using Xpert. In addition, Xpert testing had considerable incremental diagnostic yield, contributing to one quarter of all confirmed TB diagnoses. However, even with a streamlined testing process, 10% of patients with bacteriologically positive TB were not initiated on treatment, including 15% of patients diagnosed based on Xpert results. Additional strategies are needed to further reduce pre-treatment loss to follow-up (LTFU).

Our findings highlight the continued importance of sputum smear microscopy at peripheral health centers where on-site Xpert testing is currently not feasible. Sputum smear examination of a single spot specimen identified 74% of all patients with bacteriologically positive TB and led to more rapid and consistent linkage to treatment than occurred even with daily referral of samples for Xpert testing. The incremental yield of examining a second smear from the spot specimen was only 2%. This is similar to the 3% incremental yield reported previously in Uganda for single-specimen microscopy,^23^ but lower than the 11% incremental yield reported when a second smear is prepared from a second sputum specimen.^24^

In settings with access to Xpert testing, our results suggest that examination of a second smear—from the same specimen or from a second specimen—may not be necessary before referring for Xpert testing. Daily transportation of sputum samples to Xpert testing sites by *boda boda* was feasible, and the incremental yield of Xpert testing was 26%—more than twice that reported when performing smear examination of a second sputum specimen in even the most favorable evaluation.^24^ A single-specimen approach using the SIMPLE algorithm thus maximizes diagnostic yield while minimizing workload and the potential for LTFU. This approach should be considered as national guidelines begin shifting towards the utilization of Xpert for all smear-negative patients.

Our streamlined diagnostic strategy also demonstrated the potential to reduce pre-treatment LTFU. At peripheral health centers in Uganda using a routine TB diagnostic process, we found a pre-treatment LTFU rate of 24% in smear-positive patients^10^ and 90% in smear-negative patients who had samples transported to Xpert testing sites.^25^ In the present study, pre-treatment LTFU was only 8% in smear-positive patients. Moreover, 43% of smear-positive patients started treatment during their initial health center visit. Pre-treatment LTFU in smear-negative, Xpert-positive patients remained high (15%), but was substantially less than that observed in our previous study.^21^ These results strongly suggest that minimizing repeat visits to health centers to obtain a diagnosis and initiate treatment is critical for reducing patient LTFU.

Because of the pilot nature of this study, there were several potential limitations. First, it was not possible to definitively attribute the improvements in processes that were observed to our intervention. A randomized trial that includes a control group is needed to confirm these findings. Second, pre-treatment LTFU remained relatively high despite a streamlined diagnostic process. This is consistent with increasing evidence that guideline implementation requires multifaceted strategies to change behavior.^26^ In addition to a streamlined diagnostic process, future studies should evaluate interventions, such as performance feedback, to target provider behavior change and provide Xpert results by mobile telephone text messages or incentives to target patient behavior change. We are conducting a trial to evaluate on-site molecular testing with a novel platform (GeneXpert Omni; Cepheid^27^) in reducing barriers to same-day diagnosis and treatment at primary health centers. Studies assessing the costs and cost-effectiveness of single-specimen microscopy and daily sputum transportation to Xpert testing sites are ongoing.

## CONCLUSION

A streamlined diagnostic process that involves the collection of a single sputum specimen followed by on-site examination of one smear and daily referral of smear-negative specimens for Xpert testing is feasible and has strong potential to improve TB diagnosis and treatment at peripheral health centers. This approach has a higher diagnostic yield and lower pre-treatment LTFU than that reported for standard TB diagnostic evaluation. Programs should consider future operational research of this approach while awaiting the results of randomized trials.
